# Effect of Video assisted Home-Based exercise intervention on fall risk and gait parameters in older adults in India

**DOI:** 10.1093/geroni/igab046.3245

**Published:** 2021-12-17

**Authors:** Snehal Kulkarni, Aarti Nagarkar

**Affiliations:** Savitribai Phule Pune University, Pune, Maharashtra, India

## Abstract

Countries across the globe recommended isolation to protect older adults from COVID-19 infection. However, this led to decreased mobility and physical inactivity potentially increasing their risk of fall. The study was conducted in a group of 88 older adults between 60-74 years with known gait impairments and high fall risk. The participants were part of our cohort study on fall prevention program. Fall risk and gait impairments were measured using wearable sensors during the Timed-up and go test (TUG) at baseline. Using technology, a 16-week video assisted home based exercises intervention was delivered to reduce fall risk and improve gait parameters. The intervention consisted of flexibility, strengthening, balance and gait training exercises given progressively through one video session per week. The participants performed these exercises at home for the rest of the week. A home visit immediately after 16th week was arranged to collect post intervention parameters. Results showed an average 20% decrease in fall risk post intervention. An overall large effect size with Cohen’s d of 0.90 was reported for fall risk. Significant difference in TUG time (Z = -4.610, p< 0.000), stride velocity (Z= -5.035, p<0.000), stride length (Z = -5.867, p<0.000), time taken to stand (Z = -7.363, p<0.000) and time taken to turn (Z = -6.079, p<0.000) was observed in the post-test measurements as compared to pre-test measurements. Therefore, we conclude that video assisted exercise programs can be highly beneficial as alternatives to in person exercise intervention to prevent falls during COVID-19 isolation.

